# Comparison of Fecal Calprotectin Methods for Predicting Relapse of Pediatric Inflammatory Bowel Disease

**DOI:** 10.1155/2017/1450970

**Published:** 2017-04-16

**Authors:** Saranya Kittanakom, Md. Sharif Shajib, Kristine Garvie, Joceline Turner, Dan Brooks, Sufian Odeh, Robert Issenman, V. Tony Chetty, Joseph Macri, Waliul I. Khan

**Affiliations:** ^1^Department of Pathology & Molecular Medicine, McMaster University, Hamilton, ON, Canada L8S 4K1; ^2^Pathology and Laboratory Medicine, Schulich School of Medicine & Dentistry, Western University, London, ON, Canada; ^3^Farncombe Family Digestive Health Research Institute, Hamilton, ON, Canada; ^4^Hamilton Regional Laboratory Medicine Program, Hamilton Health Sciences, Hamilton, ON, Canada; ^5^Division of Pediatric Gastroenterology, McMaster University, Hamilton, ON, Canada

## Abstract

*Background. *Pediatric inflammatory bowel disease (IBD) is on the rise worldwide. Endoscopies are necessary for IBD assessment but are invasive, expensive, and inconvenient. Recently, fecal calprotectin (FCal) was proposed as a noninvasive and specific marker of gut inflammation. We evaluated the analytical performance of three FCal assays and their clinical performance in predicting relapse in pediatric IBD.* Methods. *This study used 40 pediatric IBD and 40 random non-IBD patients' fecal samples. Two automated ELISAs (Bühlmann and PhiCal® Calprotectin-EIA) and an EliA (Phadia 250 EliA-Calprotectin) were used to evaluate the analytical performance. The clinical performance was assessed by PhiCal Calprotectin-EIA, EliA-Calprotectin, and Bühlmann immunochromatographic point-of-care test (POCT).* Results.* All assays displayed acceptable analytical performance below and above the medical decision cut-off [imprecision (CV < 10% intra-assay; <15% interassay); linearity (overall mean % deviation < 16.5%)]. The agreement with PhiCal Calprotectin-EIA was 100% and 78.6% for Bühlmann (95% CI, 87.5–100; Kappa: 1) and EliA-Calprotectin (95% CI, 60.5–89.8; Kappa: 0.32), respectively, and 63.6% between Bühlmann and EliA-Calprotectin (95% CI, 46.6–77.8; Kappa: 0.16). All assays evaluated had similar clinical performance [AUC: 0.84 (EliA-Calprotectin); 0.83 (POCT and PhiCal Calprotectin-EIA)].* Conclusion.* FCal levels determined using the same method and assay together with clinical history would be a noninvasive and useful tool in monitoring pediatric IBD.

## 1. Introduction

Inflammatory bowel disease (IBD) encompasses two chronic, relapsing, life-long gastrointestinal (GI) inflammatory conditions, Crohn's disease (CD) and ulcerative colitis (UC). In 2012, it was reported that Canada has one of the highest prevalence rates and the highest incidence rate for IBD in the world [1]. Currently, the prevalence of IBD in Canada is approximately 0.7%, meaning one in every 150 Canadians has IBD [1]. The age of onset for IBD is early adulthood, but the disease may occur at any age. Canada also has one of the highest rates of childhood-onset IBD in the world [1]. The severity of IBD can significantly impact the quality of life, especially in children. They sometimes develop negative emotions and poor self-esteem resulting in troubled behavior and depression [2]. Therefore, detection and monitoring are very important in the clinical management of IBD. Timely diagnosis is particularly crucial for children, as IBD may affect their growth and sexual development [3]. However, month-long delays in diagnosis are quite common. Diagnostic criteria in IBD include nonspecific signs and symptoms such as chronic diarrhea, blood in the stool, abdominal pains, and weight loss. Several blood tests can help in the diagnosis of IBD, such as C-reactive protein (CRP), Erythrocyte Sediment Rate (ESR),* Saccharomyces cerevisiae* antibodies (ASCA), and perinuclear anti-neutrophilic cytoplasmic antibodies (p-ANCA), but none can definitively diagnose IBD. Endoscopy with biopsies is the gold standard for assessing intestinal inflammation in IBD. Endoscopy is an expensive and invasive procedure that requires a skilled operator. Furthermore, the preparatory regimen can be uncomfortable for patients. These limitations of endoscopy prevent the frequent assessment of disease activity in IBD patients.

In recent years, fecal calprotectin (FCal) has become a popular diagnostic tool in assessing IBD activity. Numerous studies have shown that the calprotectin levels detected in stool correlate well with histopathological and endoscopic findings of IBD activity [4, 5]. Calprotectin is an abundant calcium-binding protein found predominantly in neutrophils. Functions of calprotectin include inhibiting zinc-dependent enzymes by competing for zinc, potential biostatic activity against microbes through chelation of zinc ions, inducing apoptosis in malignant cells, and regulating the inflammatory process [6, 7]. The increase in FCal levels can be detected, following inflammatory damage of the intestinal mucosa and an influx of neutrophils into the intestinal lumen [8]. Calprotectin is stable in stool for up to a week at room temperature [9]. This remarkable property of FCal allows for sample collection at home by the patient and delivery to a laboratory within a few days for analysis.

FCal is a useful tool for determining the cause of abdominal discomfort, whether it is organic or functional when it is difficult to discern from symptoms or by clinical examination. An additional utility of FCal is that changes in FCal levels are a good indicator of mucosal healing. Therefore, FCal levels might be worth monitoring in patients with IBD under therapy, as well as to predict the risk of recurrence of disease prior to clinical relapse [10–16]. Calprotectin extracted from stool can be quantified using enzyme-linked immunosorbent assays (ELISAs) with the manufacturer-recommended cut-off values. This may help facilitate the diagnostic processes and may be an efficient method of monitoring IBD therapy.

A cut-off value of 50 *µ*g/g of FCal has been established for adults as well as children over the age 4 years and is used by most of the commercial ELISA kits [17]. In adults, the cut-off value of 50 *µ*g/g was found to have a 95% sensitivity and 91% specificity in differentiating IBD from healthy controls and was identified as the optimal cut-off value for detecting endoscopically active IBD [18, 19]. Children under the age of 4 tend to have higher levels of FCal, which stabilizes with age and is not gender-specific. One study recently reported three cut-off levels based on the 97.5% percentiles of FCal in different age groups, which were 538 *µ*g/g (1 < 6 months), 214 *µ*g/g (6 months < 3 years), and 75 *µ*g/g (3 < 4 years) [20].

In pediatric IBD, FCal has an excellent sensitivity for monitoring disease activity and the response to treatment [21]. Determination of FCal levels is more convenient than having repeated endoscopies. To our knowledge, this is the first Canadian study to evaluate and compare the performance of three FCal assays from different manufacturers, which is necessary given that the results of these assays may play an important role in shaping clinical decisions in pediatric IBD management. We also assessed the clinical performance of a point-of-care test (POCT) by utilizing samples from pediatric IBD patients.

## 2. Methods

### 2.1. Study Design

This retrospective study was approved by Hamilton Integrated Research Ethics Board (HiREB).

### 2.2. Participants

All stool samples were collected from consenting pediatric participants, which includes any participants up to their 18th birthday. A total of forty anonymous stool samples were randomly selected and obtained from the microbiology laboratory of Hamilton Regional Laboratory Medicine Program (HRLMP) and were used as non-IBD controls. These samples were stored at 4°C and FCal extractions were performed within 48 hours of obtaining the samples.

Eighty-nine of 125 patients attending the pediatric IBD clinic at McMaster Children's Hospital were approached and invited to participate in the study from January 2013 to July 2013, and samples were obtained from forty of the patients approached via convenience sampling. The participants had previously been diagnosed with IBD and had recently developed symptoms suggestive of relapse. The exclusion criteria for IBD patients included (1) any positive results from stool culture, ova, and parasites or* Clostridium difficile *tests, (2) inability to provide informed consent, (3) presence of serious life threatening comorbidities, (4) colectomy, (5) toxic megacolon, and (6) acute gastrointestinal bleeding. The diagnosis was made by a pediatric gastroenterologist who was unaware of FCal levels measured. The Pediatric Crohn's Disease Activity Index (PCDAI) and Pediatric Ulcerative Colitis Activity Index (PUCAI) disease activity indices were obtained prospectively for all patients with CD and UC at the time of each visit and were available for comparison [22, 23]. It is important to note here that all PCDAI scores reported exclude the height item [24]. During the colonoscopy, two biopsy specimens were obtained from the ileum and each segment of the colon in all patients. The endoscopic impression of disease activity was noted as normal or as inflamed with disease location and histological assessment by the pathologist based on standard criteria [25] was relied upon to reduce interrater variability and to assess deep tissue healing (reference standard). For patients with IBD, stool samples were collected at least 2 weeks prior to bowel preparation for a colonoscopy and neither the endoscopist nor the pathologist was made aware of the FCal levels determined.

### 2.3. Test Methods

#### 2.3.1. Fecal Calprotectin Extraction

For all assays evaluated, FCal extractions were performed within 48 hours of sample collection using the Smart Prep Extraction device from Roche Diagnostics according to respective manufacturer's instructions. Following homogenization, the extracts were centrifuged at 3000 ×g for 5 minutes; thereafter, supernatants were collected and stored at −20°C until assay. The laboratory technologist was unaware of the patient history and performed all analyses according to the manufacturer's instructions.

#### 2.3.2. Measurement of Fecal Calprotectin

Two conventional ELISA methods and a fluorescence-immunoassay (EliA) test system were used for measuring FCal levels. All three immunoassays evaluated in this study are based on the same principle of two-site sandwich technique, in which monoclonal capture antibodies highly specific to the calprotectin heterodimeric and polymeric complex are coated on the microtiter plate. For the conventional ELISAs the Bühlmann Calprotectin ELISA, extended range (30–1,800 *µ*g/g; ALPCO, Cat# 01-EK-CAL) and the PhiCal Calprotectin-EIA (Nova Century, Cat# K6927) kits were used. For the fluorescence-immunoassay method, we used the Phadia 250 EliA-Calprotectin, where ImmunoCAP 250 was the instrument (Somagen). All assays were performed according to the respective manufacturer's instructions.

Additionally, we measured FCal levels in pediatric IBD patient samples using the immunochromatographic Bühlmann point-of-care test (POCT), for which the extended range cartridge of Bühlmann POCT (30–1800 *μ*g/g; ALPCO, Cat# 01-LF-CHR25) was used according to manufacturer's instructions. Briefly, the aforementioned FCal extracts were further diluted to 1 : 150 in the extraction buffer and 80 *μ*l of the diluted samples was applied to the rapid test cartridges. Following 15 minutes of incubation at room temperature, the FCal levels were quantified using the Quantum Blue® Lateral Flow Reader (ALPCO Cat# 02-BI-POCTR-ABS).

*(a) Imprecision*. The repeatability and between-run variability of the FCal kits were assessed following Clinical and Laboratory Standards Institute (CLSI) EP05. The within-run imprecision and the between-run imprecision were evaluated (*n* = 20) at two levels of FCal. Three FCal extracts from low (non-IBD patient samples) and three from high (IBD patient samples) pools were selected for the purposes of these experiments; we additionally used controls supplied by the manufacturers. These samples were tested 20 times and the within-run imprecision was calculated. The between-run imprecision was determined by testing the samples twice a day for 10 days.

*(b) Reproducibility around (below and above) the Cut-Off*. To verify the repeatability of the FCal test results, especially around the medical decision cut-off established by the manufacturers, where FCal level below 50 *µ*g/g is considered negative and above 100 *µ*g/g is positive in IBD, we used the adopted cut-off suggested by the manufactures. These adopted values are FCal levels 20% lower than the medical decision cut-off point of a negative test (40 *µ*g/g) and 20% higher than positive test (120 *µ*g/g). Here, the pooled samples were prepared from 3 different extracts that reported FCal concentrations above (120 *μ*g/g) or below (40 *μ*g/g) the cut-off. Each pooled sample was tested 10 times to assess the reproducibility.

*(c) Linearity*. To assess the linearity over the analytical measuring range of the calprotectin assays, two fecal extract pools, low and high, were prepared from the samples based on the CLSI EP06A guidelines. The low calprotectin pool was prepared from 3 non-IBD patient samples and the high calprotectin pool was prepared from three IBD patient samples. Both pools were subjected to seven serial dilutions (e.g., pool 1 = 58, pool 2 = 335, pool 3 = 609, pool 4 = 885, pool 5 = 1161, pool 6 = 1435, and pool 7 = 1712 *µ*g/g) and were tested in triplicate. Mean percentage differences were calculated to represent the average percentage deviation between the expected and observed calprotectin concentrations across the seven dilutions of each extract using the EP Evaluator®.

*(d) Agreement between Assays*. To assess the agreement among the three assays using medical decision cut-off of below 50 *µ*g/g for FCal levels as negative, 50–100 *µ*g/g as grey zone, and above 100 *µ*g/g as positive for IBD, the forty IBD patient stool samples were evaluated.

*(e) Predicting Relapse in Pediatric IBD*. To assess the clinical cut-off of IBD relapse, FCal levels were measured using enzyme-linked immunoassays, the PhiCal Calprotectin-EIA, and the EliA assay (EliA-Calprotectin on ImmunoCAP 250), as well as using the immunochromatographic Bühlmann POCT (research study only) within 48 hours of obtaining the stool samples. As mentioned earlier, the stool sample was collected at least 2 weeks before the colonoscopy. Following the colonoscopy, the pediatric gastroenterologists' endoscopic impression of normal (negative) or inflamed (positive) bowel was made available to us.

### 2.4. Analysis

All FCal levels measured are represented as microgram of calprotectin detected per gram of fecal matter (*µ*g/g). The sample size of the study was determined based on CLSI EP9-A2 guidelines [26]. Only available data were included as part of the analysis and all statistical analyses and graphics were examined using Analyse-it® version 2.22 (Analyse-it Software Ltd., Leeds, UK) and EP Evaluator (EE) (Data Innovations, LLC, USA). The agreement between the different extraction devices, linearity, precision, and the correlation between three assays was assessed using a Bland-Altman plot, a nonparametric Passing-Bablok regression analysis, and spearman rank correlation, respectively. Indeterminate FCal values were not considered when comparing agreement between assays. The receiver operating characteristic (ROC) analyses were performed to evaluate the optimal cut-offs and to determine the FCal cut-off in evaluating IBD relapse in our study population.

## 3. Results

### 3.1. Characterization of IBD Patients and Non-IBD Patient Samples

A total of 40 pediatric IBD patients were enrolled in this study and their demographic information is presented in Table 1. The maximal extents of CD for three of the 22 patients were colonic. 11 patients with CD presented with ileocolonic disease and one patient from this group had concomitant upper GI tract involvement. Eight others had their disease restricted to the ileum, but one presented with concomitant perianal disease. In addition to that, 14 of the 22 patients with CD had a PCDAI score of less than 7.5 and 8 had scores higher than or equal to 7.5. Sixteen out of the 18 patients with UC had extensive UC and two had ulcerative proctitis. Furthermore, 14 of the 18 UC patients enrolled had a PUCAI less than 10 and the remaining 4 were equal to or greater than 10. Endoscopic description of IBD appearance correlated with the histological assessment of biopsy specimens obtained during colonoscopy. All 40 anonymous non-IBD pediatric patient samples obtained through HRLMP microbiology laboratory tested negative for* C. difficile*.

### 3.2. Imprecision

The imprecision levels for both low and high pools were adequate and acceptable for all three platforms (overall CV < 10% for intra-assay and <15% for interassay). Among the three assays, the PhiCal Calprotectin-EIA exhibited the best intrabatch precision in which the CV at low FCal level of 43 *µ*g/g is 7.6% and the CV at high FCal level of 147 *µ*g/g is 4.5% and interbatch imprecision of 6.9% at 50 *µ*g/g and 8.4% at 160 *µ*g/g (Table 2).

### 3.3. Reproducibility above and below the Medical Decision Cut-Off

Using adopted cut-offs suggested by the manufacturers, we found that of the 2 automated ELISA platforms evaluated, Bühlmann and PhiCal Calprotectin-EIA, the latter exhibited overall good calprotectin reproducibility with the CV of 8% (at 40 *µ*g/g) and 4.5% (at 120 *µ*g/g) compared to the Bühlmann (18.7% and 13.4%, resp.) automated immunoassay.

### 3.4. Linearity

In the linearity study, the observed FCal concentrations were plotted versus the expected values and the relationship was assessed using linear regression analysis (Table 3). It is noted that the analytical measuring range of FCal was stated to be up to 1,800 *µ*g/g by Bühlmann Calprotectin ELISA, up to 1970 *µ*g/g by PhiCal Calprotectin-EIA, and up to 3,000 *µ*g/g by EliA-Calprotectin. There were mean percentage deviations observed at the high concentration of FCal. Three methods were linear for the concentrations tested, which included the medical decision limit.

### 3.5. Method Comparison

**Figure 1:**
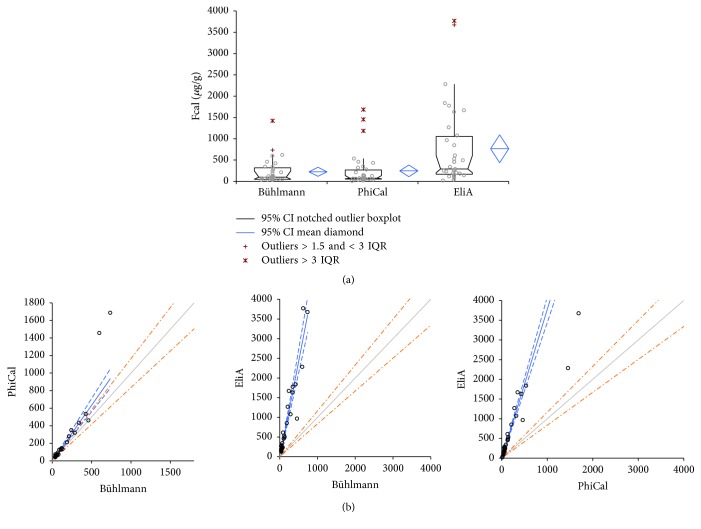
*Method comparison between three assays*. Direct comparison between the Bühlmann Calprotectin ELISA (*n* = 32; mean = 225.2; 95% CI = 119.9–330.4; SE = 51.62; SD = 292.01), PhiCal Calprotectin-EIA (*n* = 35; mean = 247.2; 95% CI = 110.0–384.4; SE = 67.516; SD = 399.432), and Phadia 250 EliA-Calprotectin (*n* = 35; mean = 765.6; 95% CI = 438.3–1092.8; SE = 161.04; SD = 952.70) using (a) descriptive comparative plot and (b) Passing-Bablok regression analysis.

To evaluate variability among the three assays, FCal of 40 stool samples was determined. The descriptive comparative plot (Figure 1(a)) represents overall higher FCal values from EliA-Calprotectin assay (mean value of 765.6 *µ*g/g) compared to the other two assays (mean FCal of 222.5 *µ*g/g for Bühlmann Calprotectin ELISA and 247.2 *µ*g/g, for PhiCal Calprotectin-EIA). The Passing-Bablok analysis showed the same finding of positive bias in the EliA-Calprotectin assay compared to Bühlmann Calprotectin ELISA and PhiCal Calprotectin-EIA (Table 4 and Figure 1(b)), especially at the higher end of the measuring range (>1,000 *µ*g/g). A good correlation between assays (*R* > 0.9) revealed both proportional and consistent differences; this is summarized in Table 4.

### 3.6. Agreement between Assays

The method comparison study exhibits the disagreement of the quantitative FCal results (Table 5). Bühlmann and PhiCal revealed very high overall agreement (100%, 95% CI, and 87.5 to 100.0%) with an excellent Cohen's Kappa coefficient. However, this agreement is not considering the discordant data in the grey zone in which six negative results and one grey zone measured by Bühlmann Calprotectin ELISA were grey zones and positive results by PhiCal Calprotectin-EIA, respectively. Bühlmann Calprotectin ELISA and EliA-Calprotectin demonstrated the least overall agreement and Kappa's coefficient. These findings implied that there is a significant difference between these two assays. PhiCal Calprotectin-EIA and EliA-Calprotectin, however, exhibited a moderate overall agreement with weak Kappa's coefficient of 0.32 (95% CI, 0.10–0.80). It is noted that the negative agreement between EliA-Calprotectin assay and either Bühlmann Calprotectin ELISA or PhiCal Calprotectin-EIA was found, indicating higher FCal results from EliA-Calprotectin (positive bias).

### 3.7. FCal in Predicting Relapse in Pediatric IBD

**Figure 2:**
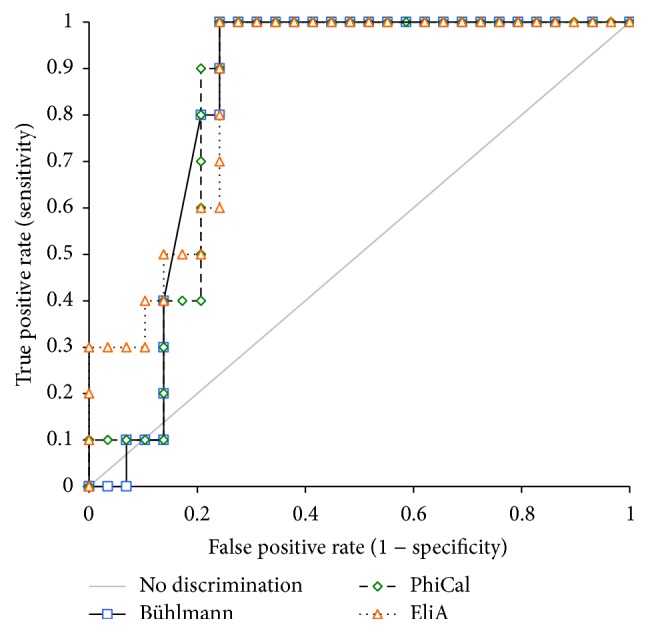
*Receiver operating characteristic (ROC) curve of fecal calprotectin (FCal)*. ROC of FCal comparing Bühlmann Calprotectin ELISA, PhiCal Calprotectin-EIA, and Phadia 250 EliA-Calprotectin with colonoscopy in 40 pediatric fecal samples. The solid line indicates values that have no discrimination. The performance of a test that exhibits the highest sensitivity and specificity shows an optimum cut-off value to evaluate the relapse of IBD.

Our data suggests that all three FCal assays have an overall equal area under the curve (AUC) of 0.83 (95% CI 0.71–0.96) (Figure 2 and Table 6). The ROC curve showed that a cut-off of 400 *µ*g/g represents the highest sensitivity at 100% (95% CI: 69.2%–100.0%) and specificity at 75.9% (95% CI: 56.5%–89.7%) for PhiCal Calprotectin-EIA. Consistent with the method comparison data, EliA-Calprotectin revealed the highest cut-off (800 *µ*g/g) to use in monitoring disease activity compared to the other two assays (400 *µ*g/g by PhiCal Calprotectin-EIA and 500 *µ*g/g by Bühlmann POCT) with a 100.0% sensitivity (95% CI: 69.2%–100.0%) and 72.4% specificity (95% CI: 52.8%–87.3%) (Table 6).

**Figure 3:**
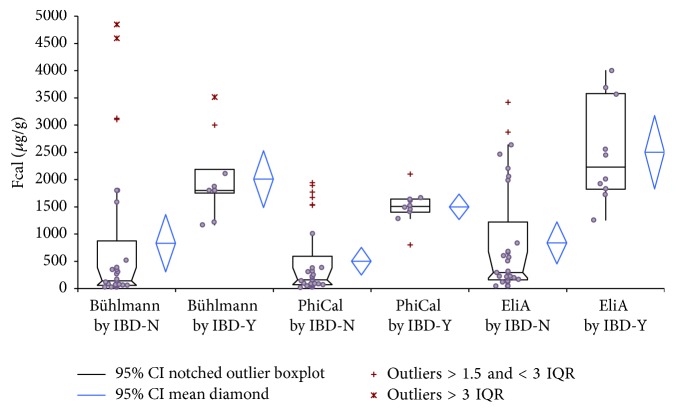
*FCal in predicting relapse in pediatric IBD patients*. FCal levels were assessed in 40 pediatric patients with IBD to evaluate the capacity of FCal to determine relapse. FCal levels (*μ*g/g) were analyzed using Bühlmann Calprotectin POCT, PhiCal Calprotectin-EIA, and Phadia 250 EliA-Calprotectin with positive (Y) or negative (N) colonoscopy of active IBD.

The cut-offs for each assay (400 *µ*g/g by PhiCal Calprotectin-EIA, 500 *µ*g/g by Bühlmann POCT, and 800 *µ*g/g by EliA-Calprotectin) were then applied to evaluate the relapse of IBD; and, at each cut-off value, IBD disease activity displayed a positive correlation with FCal levels. The Bühlmann POCT and PhiCal Calprotectin-EIA discriminated well between active and inactive IBD (Figure 3). Once again higher FCal values were reported by EliA-Calprotectin in comparison with the aforementioned two tests (Figure 3). However, this cut-off correlated with previous publications [5, 27–29]. All three assays exhibited 100% negative predictive value (95% CI ranging between 83.9% and 100.0%) and about 55.6% positive predictive value (95% CI ranging between 30.8% and 81.6%), highlighting the ability of FCal in determining relapse in this pediatric IBD population (Table 6).

## 4. Discussion

The establishment of noninvasive biomarkers is of growing interest in clinical practice, as they facilitate the objective evaluation of the disease activity and serve as a prognostic indicator of treatment outcome. Determination of FCal levels is considered to be a reliable marker of inflammatory activity in the GI tract. FCal can be used to differentiate between IBD and functional diseases like irritable bowel syndrome (IBS) [30]. In addition, it is useful in monitoring the effectiveness of IBD treatment and in detecting IBD relapses. In this study, we compared three immunoassay based FCal detection methods with a view to implement an effective and reliable procedure for the FCal test in a clinical laboratory setting.

Keeping in mind the crucial preanalytical aspects, such as stool sampling technique and transportation and storage of stool and reagents as stated by UK NEQAS external quality assurance program, herein, the performance of three immunoassays was validated and evaluated. One of the limitations of this study is the lack of a true-negative population, such as IBD patients who are not symptomatic and/or have no disease at endoscopy. We found that the PhiCal Calprotectin-EIA demonstrated the best intra- and interbatch performance. Our findings are in opposition to the findings of Whitehead et al. [31], where the authors reported that the Bühlmann Calprotectin ELISA exhibited the better interbatch assay precision. The imprecision of the three assays was comparable, but method comparisons have shown a proportional bias between assays. The linearity, however, was not as the manufacturers had stated which was demonstrated by the drift at high FCal concentrations. This drift was not reported by the previous publication [31] and this may be due to the preanalytical issues in sample extraction or analytical considerations due to the nonstandardized FCal testing. To that end, we performed several extractions with the Thermo Fisher FCal extraction device in addition to the Roche Diagnostics device (data not shown); and our findings are in line with the findings of Whitehead et al. [31] and Oyaerta et al. [32]. Both of these studies reported that the Thermo Fisher extraction devices underestimate FCal concentrations, particularly with fluid samples, as they are not as well captured by the grooves on the dosing tips as stool samples that keep their shape. It was additionally reported that FCal extracts from this device yield higher values when measured using the EliA method. We made similar observations; however, the Thermo Fisher extraction kit may be a good alternative to the Smart Prep by Roche Diagnostics due to its ease of use.

Bühlmann Calprotectin ELISA and PhiCal Calprotectin-EIA have the closest relationship for cross-kit variation study and, EliA-Calprotectin showed the highest FCal read-out with very good precision (imprecision studies), supporting the findings of a previous publication [31]. Moreover, the quantitative agreement between three assays suggested that the differences between Bühlmann Calprotectin ELISA and EliA-Calprotectin could be due to the different antibodies used, which may recognize different epitopes of the heteroduplex of calprotectin. Our findings demonstrate that the FCal levels measured by different assays are not interchangeable and laboratories should state the methods used.

The medical decision cut-off of FCal has been proposed by many studies [18, 30, 31, 33, 34] with an overall good sensitivity and specificity in differentiating IBD from other forms of inflammation. The clinical usefulness of calprotectin and cut-off values has been intensively studied, including, in the diagnosis and differential diagnosis of IBD, disease activity monitoring, disease course, and effect of treatment [35, 36]. Chung-Faye et al. [37] and Carroccio et al. [38] used significantly different cut-offs of 25 *µ*g/g and 170 *µ*g/g in their studies, respectively. In IBD relapse prediction, the study by Tibble et al. [39] had applied FCal > 50 *µ*g/g in adults patients, whereas Walkiewicz et al. used the cut-off as high as >400 *µ*g/g of FCal in pediatric IBD population [28]. Though many studies have suggested different cut-off values, which take into account the type of assay used and the population that the tests were applied to, Waugh et al.'s suggestion of using a cut-off value of 50 *µ*g/g FCal is reasonably sensitive and specific [30]. A greater accuracy was found when using an FCal cut-off value of 100 *µ*g/g instead of 50 *µ*g/g in the diagnosis of IBD [40]. Using a cut-off of 250 *µ*g/g, the sensitivity and specificity of detecting disease activity were found to be 90% and 75%, respectively, for one study [35] and 80% and 82%, respectively, in other similar studies [40, 41]. In addition to that, an accuracy of 88% and 74% with a cut-off value of 250 *µ*g/g in detecting disease activity of UC and CD, respectively, was reported [42]. Based on our results of the ROC curve, the optimal FCal cut-off point to differentiate active from inactive IBD in pediatric patients was 400, 500, and 800 *µ*g/g measured by PhiCal Calprotectin-EIA, Bühlmann POCT, and EliA-Calprotectin, respectively, and this is in agreement with previous studies [5]. With the above-selected cut-off in predicting relapse, the sensitivity of 100% and specificity of 75.9% were achieved. Our data also showed a 100% negative predictive value for all assays evaluated, which emphasizes the potential usefulness of FCal in eliminating unnecessary endoscopic evaluations of patients with negative FCal results. It is noted that the optimal medical decision cut-off point for monitoring and predicting the recurrence of pediatric IBD is not yet clear, but significantly higher values have been reported [5]. However, FCal levels > 500 *µ*g/g have been proposed as the cut-off point for adult IBD patients as it correlates well with disease activity. This emphasizes the need for standardization of FCal assays, as well as a well-defined cut-off point for its clinical utility, especially FCal levels ranging within 51–99 *µ*g/g (the grey zone) to abolish the interpretive ambiguity.

The other important factors which are preanalytical, such as sample collection, disease activity, treatment, and specific method used, as well as FCal stability, consistency of sample analyzed (firm or loose stool), sample storage, and the freeze-thaw cycle of the sample, may considerably influence the FCal level measured. Therefore, the medical decision cut-off should be established by taking these factors into account. Dhaliwal et al. reported that FCal levels decrease significantly when the extract is stored at room temperature, whereas extracts stored at −20°C remain stable for up to two and a half months [40].

In our study, the Bühlmann POCT showed as good and comparable sensitivity as the automated platforms, PhiCal Calprotectin-EIA and EliA-Calprotectin. The introduction of the automated platform in our facility led to flexibility reduced cost and improved quality of service. Given the good performance characteristics of the automated platforms, they can be used as the first screening tool in patients with suspected IBD but with a different cut-off point in mind. Our findings are in agreement with others [43] and demonstrate that determination of FCal levels may be useful in avoiding the unnecessary, undesirable, and invasive procedure such as endoscopy and/or selecting the best treatment plan especially for children with IBD. FCal testing has the potential to become a clinically relevant tool in determining whether patients have IBD and even predicting relapse in IBD patients, in consideration with the limitations of assay/platform as well as their differences. Regardless of the grey zone and differing cut-off values reported, the clinical interpretation of FCal results should be made with caution depending on the assay used. Therefore, we highly recommended the use of the same test by the same manufacturer during follow-ups or monitoring of the treatment.

## Figures and Tables

**Table 1:** Pediatric IBD patient demographic information.

	Crohn's disease	Ulcerative colitis
Number of participants	22	18
Mean age (range) in years	14.6 (11–17)	14.1 (11–17)
% female	45.5	33.3
Mean disease duration (range) in years	2.8 (0.33–7)	2.3 (0.66–9)

**Table 2:** Studied imprecision.

Manufacturer	Within run (*n* = 20)				Between run (*n* = 20)	
	Mean	SD	95% CI	% CV	Mean	% CV
Bühlmann						
Low sample	58	4.7	3.6–7.1	8.1	38	9.1
High sample	396	15.1	11.3–22.1	3.8	408	12.4
PhiCal						
Low sample	43	3.3	2.3–5.9	7.6	50	6.9
High sample	147	6.7	4.6–12.2	4.5	160	8.4
EliA						
Low sample	64	2.5	1.8–4.2	3.9	42	7.5
High sample	342	15.6	11.9–22.8	4.6	640	6.5

**Table 3:** The linearity of dilution.

Manufacturer	Slope	Intercept	Overall mean % deviation
Bühlmann	0.87 (0.85 to 0.89)	4.1 (−2.6 to 10.9)	9.9
PhiCal	1.24 (1.16 to 1.31)	−1.8 (−9.4 to 5.6)	16.5
EliA	1.11 (1.07 to 1.16)	−1.3 (−4.9 to 2.2)	11.1

**Table 4:** Summary of assay performance (Passing-Bablok).

Bühlmann				
PhiCal	30	1.3 (1.1 to 1.4)	−4.4 (−16.0 to 8.1)	0.94 (0.87 to 0.97)
EliA	30	5.0 (4.4 to 5.6)	−56.0 (−108.3 to −4.0)	0.94 (0.88 to 0.97)
PhiCal				
EliA	33	3.8 (3.5 to 4.1)	−21.0 (−44.3 to −0.4)	0.93 (0.87 to 0.97)

**Table 5:** Agreement between assays.

Comparison	*N*	Cohen's Kappa	Positive agreement	Negative agreement	Overall agreement
Bühlmann					
PhiCal	27	1 (1 to 1)	100%	100%	100.0% (87.5 to 100.0%)
EliA	33	0.16 (0 to 0.54)	100%	14%	63.6% (46.6 to 77.8%)
PhiCal					
EliA	28	0.32 (0 to 0.80)	100%	25%	78.6% (60.5 to 89.8%)

**Table 6:** Clinical performance of FCal.

Platform	AUC	95% CI	Cut-off (*µ*g/g)	Sensitivity (%)	Specificity (%)	Positive predictive value (%)	Negative predictive value (%)
PhiCal	0.83	0.71 to 0.96	400	100.0 (69.2–100.0)	75.9 (56.5–89.7)	58.8 (32.9–81.6)	100.0 (84.6–100.0)
EliA	0.86	0.74 to 0.98	800	100.0 (69.2–100.0)	72.4 (52.8–87.3)	55.6 (30.8–78.5)	100.0 (83.9–100.0)
Bühlmann (POCT)	0.83	0.70 to 0.96	500	100.0 (69.2–100.0)	72.4 (52.8–87.3)	55.6 (30.8–78.5)	100.0 (83.9–100.0)
